# Single-trial dynamics explain magnitude sensitive decision making

**DOI:** 10.1186/s12868-018-0457-5

**Published:** 2018-09-10

**Authors:** Angelo Pirrone, Wen Wen, Sheng Li

**Affiliations:** 1 School of Psychological and Cognitive Sciences, Peking University, Beijing, 100871 China; 2 Beijing Key Laboratory of Behavior and Mental Health, Peking University, Beijing, 100871 China; 3 PKU-IDG/McGovern Institute for Brain Research, Peking University, Beijing, 100871 China; 4 Key Laboratory of Machine Perception (Ministry of Education), Peking University, Beijing, 100871 China

**Keywords:** Magnitude sensitivity, Decision making, Decision boundary, Reward, DDM

## Abstract

**Background:**

Previous research has reported or predicted, on the basis of theoretical and computational work, magnitude sensitive reaction times. Magnitude sensitivity can arise (1) as a function of single-trial dynamics and/or (2) as recent computational work has suggested, while single-trial dynamics may be magnitude insensitive, magnitude sensitivity could arise as a function of overall reward received which in turn affects the speed at which decision boundaries collapse, allowing faster responses as the overall reward received increases.

**Results:**

Here, we review previous theoretical and empirical results and we present new evidence for magnitude sensitivity arising as a function of single-trial dynamics.

**Conclusions:**

The result of magnitude sensitive reaction times reported is not compatible with single-trial magnitude insensitive models, such as the statistically optimal drift diffusion model.

## Background

Many types of decisions cannot be described by the typical accuracy-based experimental setting adopted to study decision making, in which subjects are rewarded only on making a correct choice, or receive a fixed reward at the end of the experiment. This is for example the case of pervasive value-based decisions; in this case subjects are rewarded by the value of the chosen alternative, and not only on making a correct choice. Furthermore, in many ecological settings, a fast but potentially inaccurate decision is to be preferred over a slow but accurate one [1–3]. With regards to value-based decisions, this is the case in which alternatives are perishable, or when spending time in making a decision can result in competition with other agents. In such cases there is a time cost for additional time spent in making a decision. When the difference between two alternatives is ‘high’, the decision is driven in a bottom-up fashion by the difference between the two alternatives and the most valuable one is chosen. However, the insight behind previous work is that, in order to avoid decision deadlocks over difficult decisions, decision makers adopt a speed-value trade-off: sacrifice negligible accuracy with fast and potentially inaccurate decisions [1–3]. Following this rationale, as the magnitude of the alternatives increases, decisions are predicted to be made faster [1–3].

Interestingly, empirical evidence for a speed-value trade-off has been provided [2, 3]. For example, it has been found [3] that both humans performing a perceptual decision making task, and monkeys performing a value-based decision making task, were faster in making decisions over two equal alternatives as the magnitude of the alternatives increased. This means that, although the difference between alternatives was zero across all conditions of interest (but also unequal alternatives were presented during the experiments), as the overall magnitude increased, decisions were made faster. This result is relevant for models of choice such as the drift diffusion model (DDM; [4]), which by accumulating only difference in evidence, cannot accommodate the result of decreasing reaction times (RTs) for equal alternatives of increasing magnitude. In the experiments testing humans, the overall reward that subjects received for any given trial was fixed [2] or subjects were performing the task in exchange of course credits [3] and reward received was not correlated with the overall magnitude of the alternatives. This result seems to suggest that the observed magnitude sensitivity was not related to overall reward received but it was instead a property of the decision making process itself, arising from a single-trial magnitude-sensitive decision architecture [2, 5–8]. In particular, it has been demonstrated [2] that two models can exhibit magnitude sensitive decision making: a DDM with magnitude dependent noise in which higher magnitude results in higher noise, and the Leaky Competing Accumulator (LCA, [9]), an ‘hybrid’ model of decision making which is sensitive to both difference and absolute input values and that can be conceptualized as a DDM which at the initial stages of accumulation is dominated by magnitude sensitivity, which affects decision thresholds, and at the later stages of accumulation is instead dominated by the difference in evidence between the alternatives [10].

Recently, the optimal policy for value-based decision making has been derived [11], and it has been shown that such policy is implemented by a variant of the DDM with boundaries that collapse over time during a trial. The slope of the boundary is determined by the utility function that subjects receive; when the utility function that subjects receive is linear, the slope of the boundary is one, meaning that boundaries are parallel. However, it has been demonstrated [11] that when the utility function is not linear or when prior/likelihood distributions of reward/evidence are correlated in particular ways (for details see [11]), the slope of the boundary ceases to be one and the boundaries are not parallel. In their formal account of decision making, authors also derived the optimal policy for accuracy-based decision making, and showed that this is implemented similarly to the case of value-based decision making. Although value and accuracy-based decision making have been investigated using DDMs (for accuracy-based examples see [4]; for value based examples, see [12–14]), the work investigating the optimal policy for value and accuracy-based decisions highlights important qualitative differences between accuracy and value based-decision making. First, in value-based decision making, thresholds collapse more rapidly compared to accuracy-based decisions. Second, in value-based decision making, thresholds collapse more rapidly as the total reward that subjects receive increases, while the optimal policy for accuracy-based decision making is insensitive to total reward received [11].

An important difference with previous magnitude sensitive accounts [1–3] is that in the optimal policy [11], magnitude sensitivity arises as a function of overall reward received while in previous accounts [1–3] magnitude sensitivity is implemented as a function of single-trial magnitude. While the former account seems to suggest a ‘top-down’ strategic adjustment of decision criteria, dependent upon overall reward received (since subjects need to maintain a memory trace of overall reward received), in the latter account, magnitude sensitivity arises spontaneously in a ‘bottom-up’ fashion and is trial-by-trial dependent (since the decision is exclusively driven by the features of the stimulus presented).

Inspired by the literature presented above, here we directly test contrasting explanations for magnitude sensitivity; in particular we test for the first time whether magnitude sensitivity is dependent upon single-trial dynamics and/or upon overall reward received. Furthermore, to strengthen previous results [3], we attempt to provide further evidence for magnitude sensitivity for equal alternatives, using a different experimental paradigm.

Testing different explanations for magnitude sensitivity can be done by directly comparing goodness of fit of contrasting models, or by designing behavioral experiments for which different models make different predictions. Here, we use a simple behavioral experiment that allows us to test predictions regarding magnitude sensitivity at the single-trial level and at the overall reward level, and that allows us to test a family of models that make different behavioral predictions; in particular, single-trial magnitude sensitive models (e.g., [2, 5]) predict single-trial magnitude sensitivity but do not make specific predictions regarding an additive effect of overall reward received in affecting decision thresholds, while the optimal account [11] predicts single-trial magnitude insensitive RTs for equal alternatives and is affected by overall reward received. Together with a simple experiment, we review existing theoretical arguments and empirical results regarding magnitude sensitivity in decision making.

In our experiment, subjects were presented with two numerosity stimuli; array of dots presented to the left and right of a computer screen. In the accuracy-based condition, subjects were rewarded on making a correct choice (i.e., are there more dots on the left or on the right?), while in the value-based condition, subjects had to choose which of the two stimuli they wanted to collect and were paid every 1000 dots they collected. There was not a correct or wrong choice in the value-based session, meaning that subjects were rewarded by the value of the chosen alternative, even if the lower value alternative was selected. Furthermore, for both sessions we created high and low reward scenarios, by manipulating the reward that subjects received during the accuracy session and the total number of dots that subjects could gain in the value based session. All blocks were blocked by duration, rather than by number of trials and this resulted in a time cost associated with longer decisions. Together with the overall reward received, we also manipulated single-trial magnitude conditions, hence our study included (1) accuracy-based versus value-based scenarios, (2) high versus low overall reward received and (3) a continuum of equal alternatives of increasing magnitude that was constant across scenarios and overall reward received.

Also here, as done previously [3], although subjects were presented with both equal and unequal alternatives, our focus is exclusively on equal alternatives. Using unequal alternatives, when the overall magnitude is increased while keeping the difference between the two alternatives constant, also the discriminability between the two stimuli is changed, according to well known psychophysical transformations. For example, judging a 1 cm length difference between two stimuli having an average length of 5 cm is not psychologically comparable to judging a 1 cm difference in length between two stimuli having an average length of 100 cm. Although the same physical difference between the two stimuli is maintained, the two decisions are not psychologically directly comparable with regards to perceived difference between the stimuli. At the same time, it is not possible to define a priori and across-subjects an equally perceived difference between two conditions consisting of unequal alternatives. With equal alternatives, the same perceived difference between the alternatives (i.e., zero) is instead maintained constant when the overall magnitude is varied. For this reason, equal alternatives allow to test predictions regarding magnitude sensitivity excluding perceptual confounds that instead do affect unequal alternatives whenever the overall magnitude is manipulated. However, presenting exclusively equal alternatives would result in a nonsensical experiment, hence the necessity of mixing our conditions of interest, equal alternatives, with unequal alternatives for which it is possible to define a correct and a wrong response.

## Methods

### Participants

Twenty-one participants (11 females) voluntarily took part in the experiment and were rewarded monetarily on the basis of their performance (details for the reward scheme are reported below). Their mean age was 23 years, and ranged from 20 to 27 years. Subjects were healthy university students and had normal or corrected-to-normal vision. The study received ethical approval from the Departmental ethics committee and informed consent was obtained from all subjects.

### Stimuli and procedure

The stimuli were presented, using PsychoPy [15], on a 36 × 27 cm CRT screen with a refresh rate of 100 Hz at a viewing distance of 57 cm, where the head of the subject was positioned on a chin rest.

**Fig. 1:**
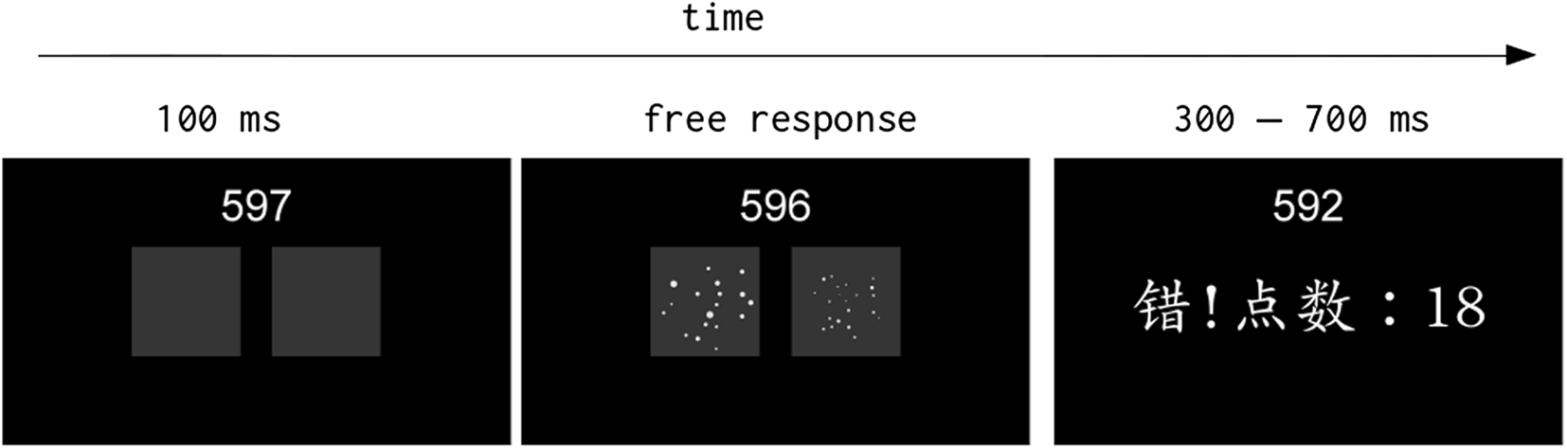
Stimulus example and trial sequence for the training phase. The number on top indicates the seconds left before the block is going to finish. After 100 ms, during which participants were presented a blank screen, the two dots array were presented. In the specific example, 18 versus 20 dots are presented. Subjects could make a response in their own time. After giving a response, during the training subjects were presented with visual feedback for a duration that could randomly vary between 300 and 700 ms. The feedback (not to scale in this example) shows a training trial during which participants were shown if they were correct or wrong (wrong in the specific example) and the number of dots collected. In the accuracy block only the accuracy feedback was provided and if subjects were correct the feedback was presented for 300 ms otherwise if they were wrong the feedback was presented for 1000 ms. In the value-based session only the information regarding the number of dots collected was provided for a random duration between 300 and 700 ms

At 6.5$^\circ$ on the left and on the right from the center of the screen subjects were presented with the two arrays of dots. Such stimuli were previously generated using code made freely available [16] which allows to generate a set of stimuli in which the continuous variables associated with numerosity (e.g., area occupied by the stimulus, dot diameter etc) are randomly varied across stimuli. For example, for a stimulus consisting of 12 versus 10 dots, the area occupied by the 10 dots array could be either smaller or bigger than the area occupied by the 12 dots array. Each array of dots was drawn within a 10 by 10$^\circ$ image, and subsequently presented on screen during the experiment. For each condition multiple array combinations were generated (i.e., subjects were not presented one stimulus per condition throughout the experiment). Stimuli values are reported in Table 1, while a stimulus example and trial sequence is reported in Fig. 1. Stimuli consisted of both equal (i.e., same number of dots on the left and on the right) and unequal alternatives.

**Table 1:** Stimuli values and probability, averaged across participants, that a specific stimulus pair was displayed during a high reward (HR) block or a low reward (LR) block

Dot pairs	HR probability	LR probability
Equal alternatives (12, 18, 24, 30 or 36 dots)	0.040	0.040
12 vs 10 OR 12 vs 14	0.068	0.108
18 vs 15 OR 18 vs 21	0.061	0.089
24 vs 20 OR 24 vs 28	0.075	0.074
30 vs 25 OR 30 vs 35	0.088	0.062
36 vs 30 OR 36 vs 42	0.108	0.067

Conditions of interest, equal alternatives, always had the same probability of being presented on screen, across sessions (accuracy versus value) and overall reward received (high versus low). In particular, each pair of equal alternatives (e.g., 12 versus 12 dots, 18 versus 18 dots etc) had a probability of .04 of being presented, for a total of 20% of equal alternatives presented during the experiment

The experiment consisted of two different sessions, the accuracy-based session and the value-based session, and of two types of rewards, high and low reward. In the value-based session, subjects gained 1 yuan every 1000 dots they collected. There was no correct or wrong response, meaning that if subjects chose the array with less dots, they were still rewarded by the number of chosen dots. In the high-reward session, stimuli with more dots had a higher probability of being presented, hence subjects would on average gain more reward. Conversely, for the low-reward condition, stimuli with fewer dots had a higher probability of being presented during each block. The probability of a dot pair to be presented for the high and low reward conditions is reported in Table 1. Subjects were explicitly instructed not to count the dots, and performed four 10-min blocks. After each block they could take a self-paced break. For the value-based session, the four sessions could randomly consist of two high and then two low-reward conditions or vice versa (the order was counterbalanced). After each trial subjects were presented the number of dots collected for a time that could randomly vary between .3 and .7 s, after which a new trial was presented.

For the high-reward accuracy condition subjects were rewarded .08 yuan for a correct response; for the low-reward accuracy condition subjects were instead rewarded .04 yuan for a correct response. If subjects were correct, they were presented ‘correct’ for .3 s, while if they were wrong they were presented ‘wrong’ for 1 s. Hence, there was a time cost associated with a wrong response. It was necessary to introduce a time cost, since it has been shown [10] that in a task in which the total block duration is fixed, and for which there is no time penalty for a wrong response, the optimal strategy is answering randomly as fast as possible. Also in this case, subjects performed four 10-min blocks. For the accuracy-based session, the four sessions could randomly consist of two high and then two low-reward conditions or vice versa. Each block had a probability of a dot pair to be presented as reported in Table 1; however there was no relation between block reward and probability of a dot pair to be selected (i.e., as there was indeed for the value-based session). For the accuracy-based session, the accuracy feedback for equal alternatives was randomly determined as correct or wrong.

For both the value and accuracy based condition, at 9$^\circ$ from the center of the screen subjects were presented 4$^\circ$ white digits counting down from 600 to 0 (i.e., 10 min = 600 s). When the time finished, subjects were presented ‘time finished’ for 500 ms.

For both the accuracy-based and value-based sessions there was no relation between trial magnitude and trial number, meaning that different magnitude conditions were presented randomly.

Subjects performed the accuracy and the value-based sessions in random order (counterbalanced across subjects).

Before the experiment started, subjects performed a single 10 min session to familiarize with the trials. Here, they were instructed to decide whether there were more dots on the left or on the right and after each trial they were presented, for a random duration between .3 and .7 s, if they were correct or wrong and how many dots they selected for the specific trial. For the training, the probability of a dot pair to be presented was constant across conditions.

## Results

### Behavioral analyses

We specifically focused on conditions of interest, equal alternatives. Regarding equal alternatives, no data were excluded from the analyses. We ran a repeated measures ANOVA and we included the following three factors and all possible interactions: session (accuracy-based versus value-based), overall reward received (high vs low) and trial magnitude (12, 18, 24, 30 or 36 dots). Analyses based on log-transformed RTs yielded similar results, hence for simplicity and ease of communication we focus on the analyses on raw RTs.

**Fig. 2:**
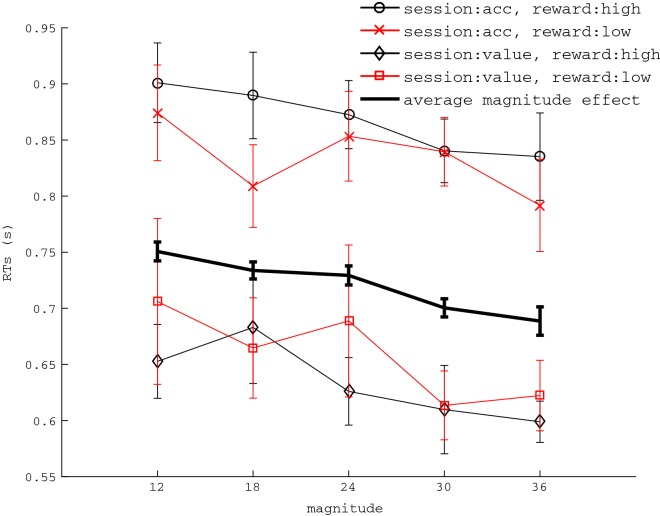
Effects of session and magnitude on RTs of equal alternatives. Overall reward received did not affect RTs. The thicker line represents the main effect of magnitude. Error bars represent standard error of the mean

For equal alternatives, as can be seen from Fig. 2, session affected mean RTs, F(1,20) = 11.16, p= .003, $\eta ^{2} = .358$, but overall reward received did not (*p* = .819). A further significant effect was that of trial magnitude, Fig. 2, F(4,80) = 5.05, $p <.001$, $\eta ^{2} = .216$, showing that RTs decreased as magnitude increased. A post-hoc mixed effect regression of trial magnitude on mean RTs for which we included random slopes and intercepts confirmed that RTs significantly decreased as magnitude increased, b = − .003 (95% CI − .004, − .001), $p < .001$. All other main effects and interactions did not reach significance, $p > .16$. For none of the magnitude conditions, participants had a probability of choosing left over right higher than chance (all $p > .1$), showing that subjects were not biased in choosing one alternative over the other.

**Fig. 3:**
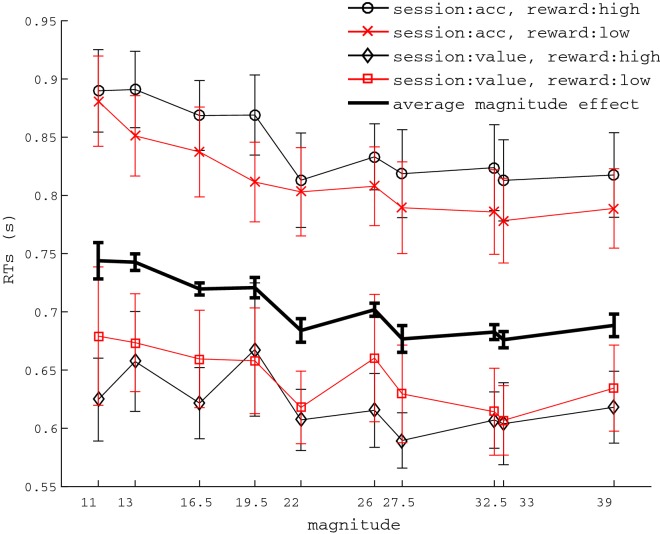
Effects of session and magnitude on RTs of unequal alternatives. Overall reward received did not affect RTs. The thicker line represents the main effect of magnitude. Error bars represent standard error of the mean

Although our focus is on equal alternatives, here we also report results for unequal alternatives. When only unequal alternatives were considered, the exact same pattern reported for equal alternatives was replicated for RTs. In particular, as shown in Fig. 3, session affected mean RTs (mean difference between the two sessions = .196 s), F(1,20) = 9.457, p= .0006, $\eta ^{2} = .321$, but overall reward received did not (*p* = .805). Trial magnitude affected RTs, Fig. 3, F(9,180) = 7.119, $p <.001$, $\eta ^{2} = .262$. A mixed effect regression for magnitude on mean RTs resulted significant, b = − .002 (95% CI − .003, − .001), $p < .001$.

**Fig. 4:**
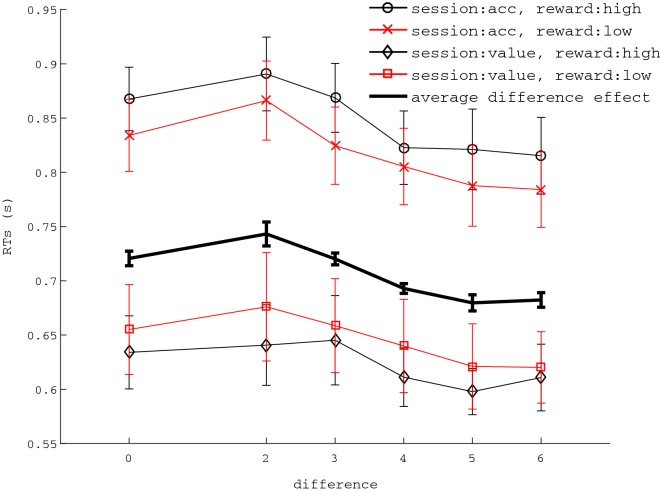
Effects of session and difference on RTs of unequal alternatives. Overall reward received did not affect RTs. The thicker line represents the main effect of difference. Error bars represent standard error of the mean

When data were analyzed with regards to difference, Fig. 4, RTs differed between sessions F(1,20) = 9.851, *p* = .005, $\eta ^{2} = .330$ with a mean difference of .198 s between the slower accuracy-based session and the faster value-based session. Difference affected RTs, F(5, 100) = 9.824, $p<.001$, $\eta ^{2} = .329$, showing that RTs decreased when difference increased. A mixed effect regression for difference on mean RTs resulted significant, b = − .009 (95% CI − .01, − .005), $p < .001$. Note, as we have shown, magnitude affects RTs, and equal alternatives had a mean magnitude of 24. The effect of magnitude might render such conditions slightly faster (although not significantly) than conditions having a difference of 2 and for which overall magnitude was 12. This pattern suggests that the effect on RTs driven by doubling magnitude from 12 to 24 dots may be stronger than the effect on RTs driven by a difference of 0 versus 2 dots.

### Model fitting

One model that could account for magnitude sensitivity at the single trial level is the LCA [9], which can be approximated by a DDM [4] with magnitude sensitive decision thresholds. The LCA can be conceptualized as a DDM that at the early stages of evidence accumulation is magnitude sensitive and that at the later stages of evidence accumulation is instead dominated by difference between the alternatives [10]. We reasoned that if we fit a DDM with magnitude sensitive boundary separation for the equal alternatives, for which difference in evidence/value could not have played any role, we should find the estimated boundary to significantly differ across magnitude conditions, and this would provide further proof for magnitude sensitivity at the single-trial level. Note however that in the canonical DDM [4], the boundary separation is not stimulus dependent and it is generally set before stimulus appearance.

The parameters that define the DDM are (1) the boundary separation, which reflects the speed-accuracy trade-off adopted by subjects and their response conservativeness (2) the non-decision time, which reflects the time to encode the stimulus and execute the motor response, (3) across-trial variability in non-decision time, (4) the drift rate, which reflects the information carried by the stimulus which in our case it is null, (5) across-trial variability in drift rate, (6) the bias for a response which represents a priori commitment towards one of the two alternatives and (7) its across-trial variability.

In order to estimate the DDM parameters we used DMAT [17] for Matlab. Using the options provided by DMAT, we opted for a chi-square minimization fitting routine of the data represented by five different bin edges for ‘left’ and ‘right’ responses. We fitted a constrained model in which each session was fitted separately for each subject. Given that reward did not affect our behavioral data, we collapsed conditions across reward. The model that we fitted allowed the boundary to be a linear function of trial magnitude, while all other parameters were kept constant within each session.

A table of estimated parameters is reported in Table 2. We run Wilcoxon-signed rank tests on the estimated parameters. If boundary was not affected by magnitude, we should expect the slope of the boundary not to differ from zero for both sessions. However, for both sessions, the slope of the boundary was significantly lower than zero (M = − .019, SD = .054, *p* = .041 for the accuracy session and M = − .044, SD = .097, *p* = .012 for the value session), showing that the boundary significantly decreased when single-trial magnitude increased. The difference between the two slopes was not significant (*p* = .838). The intercept of the two boundaries differed significantly between sessions (*p* = .011), and it was higher for the accuracy than for the value session (M = .166, SD = .063 for the accuracy session and M = .15, SD = .11 for the value session), confirming once again that subjects were more cautious for the accuracy-based session compared to the value-based session.

Regarding the bias, it did not differ from the unbiased level for both sessions ($p >.89$) and it did not differ between sessions ($p \sim 1$). However, the difference in variability in bias between the two sessions was marginally significant (*p* = .046) and higher in the accuracy than in the value session (M = .062, SD = .06 for the accuracy session and M = .039, SD = .052 for the value session).

As expected subjects had a higher non-decision time for the accuracy session (M = .468, SD = .212) than for the value based session (M = .365, SD = .329, *p* = .022), suggesting that between sessions subjects decreased their time to execute the motor response. The across trial variability in non decision time did not vary significantly between sessions (*p* = .374).

As expected the drift rate did not vary between sessions (*p* = .272) and was indistinguishable from zero ($p > .275$). However, the parameter capturing across-trial variability in drift rate, differed between sessions (*p* = .02) and was lower in the accuracy than in the value session (M = .179, SD = .163 for the accuracy sesssion and M = .279, SD = .154 for the value session). Across-trial variability in drift rate can be interpreted as variation in attention/motivation [17]. This result suggests that, in line with our hypotheses, subjects were paying less attention to the stimuli in the value-based session compared to the accuracy-based session.

In order to investigate the goodness of fit of our model, a chi-square test tested the discrepancy between predicted and observed quantile RTs; 76% of all datasets had a lower value than the critical value of a chi square at $p < .05$ with degrees of freedom equal to the number of degrees of freedom in the data minus the number of parameters of the model. This result confirms that the model can account for the data well.

**Table 2:** Table of estimated parameters for the accuracy-based and value-based session

Session	Bounary intercept	Boundary slope	Ndt	Var. drift	Bias	Var. bias	Var. ndt	Drift
Accuracy	.166	− .019	.468	.179	.506	.062	.275	− .005
Value	.150	− .044	.365	.279	.495	.039	.340	− .024

‘Ndt’ stands for ‘non-decision time’, while ‘var’ stands for ‘variability’. Statistical tests comparing the two sessions are reported in the main text

## Discussion

Here, we have investigated whether magnitude sensitivity arises as a consequence of single-trial magnitude sensitivity and whether overall reward received results in threshold adjustments, allowing faster RTs as the overall reward received increases [11]. In addition, we attempted to replicate the result of magnitude sensitivity in human decision making [2, 3] using a different experimental paradigm. In our experiment we manipulated the reward structure, value-based versus accuracy-based, the overall amount of reward that subjects received, high versus low, and the single trial magnitude. Our results show (1) a replication of previous results showing magnitude sensitive RTs for equal alternatives in decision making [2, 3], (2) single-trial magnitude modulates RTs while overall reward received, in our task, did not affect RTs.

Together with previous studies [2, 3] in which it is important to recall that overall reward was fixed across trials, our results seem to challenge the prediction of the optimal account [11]. In the optimal account, the dynamics of a single trial are dominated by difference in evidence alone, while it is overall reward received to affect the speed at which boundaries decrease. Furthermore, in the optimal account [11], the result of magnitude sensitivity for equal alternatives is expected only when decision boundaries are not parallel (in [18] an in-depth discussion regarding this issue is provided); the optimal account with parallel-collapsing boundaries cannot accommodate magnitude sensitive RTs for equal alternatives since single-trial dynamics are driven by input difference alone, hence all equal alternatives are predicted to have same RTs since input difference is constant and equal to zero.

A further factor to consider when comparing the optimal magnitude-sensitive account [11] and the single-trial magnitude sensitive accounts is that, in the former, subjects should know the reward structure of the task in order to adjust their decision thresholds. As discussed in a previous theoretical paper [1], in many naturalistic settings, subjects do not have access to such information; hence a mechanism that spontaneously exhibit trial-by-trial magnitude sensitive behavior without knowledge of the reward structure explains magnitude sensitive decision making more parsimoniously compared to a mechanism that requires a priori, trial-by-trial updated knowledge of the reward structure and of the overall reward of a specific task. Our modeling results show that trial magnitude affects the decision boundary and this in turn results in faster and potentially less accurate decisions.

Here, we did not find support for the role of total reward received in decreasing decision thresholds, not for accuracy-based nor for value-based decisions. We are cautious in interpreting negative results and we do not take this result as compelling evidence that overall reward received does not affect threshold adjustment in simple value-based and accuracy-based decisions. It is possible that the difference in reward between the two sessions might not have motivated subjects towards decreasing the threshold further in order to accumulate more reward. Another factor to consider is that, in the low reward value-based session subjects had a mean RT of about .650 s; recall that subjects had to estimate two separate stimuli and it is possible that the mean RT of .650 s is already the asymptotic RT for such type of decisions, meaning that no manipulation could have decreased the boundary separation further. Also, it is possible that the specific type of stimuli that we have used might not have elicited the expected result; future investigations could use intrinsically valued stimuli, such as food images, in order to elicit the phenomenon of interest.

However, using intrinsically valued stimuli is problematic for the presentation of equal alternatives, and for the manipulation of difference and magnitude. Furthermore, the operationalization itself of intrinsically valuable stimuli is particularly noisy since it relies on tools such as Likert scales (e.g., in order to make a judgment regarding the value of a specific stimulus such as an image representing food). With regards to our hypotheses, our approach allowed us a thorough control of possible confounds and a better operationalisation of difference, magnitude and overall reward which were dependent upon physical features of the stimuli. Furthermore, a crucial factor to point out is that, regardless of an additive role of total reward in magnitude sensitivity, our results, together with previous results showing magnitude sensitivity for equal alternatives [3], show that a single-trial purely relative decision making model alone, such as that proposed by the optimal account [11] or the canonical DDM [4] in which the boundary separation is fixed across magnitude conditions, cannot accommodate the magnitude sensitivity that we have observed. Our result can be accounted by magnitude sensitive models such as the LCA [9], as our model fitting suggests by showing magnitude sensitive decision boundaries. However, we do not exclude other theoretical explanations. Recent research has shown the presence of magnitude dependent noise [19, 20] and it has been shown [2] that a DDM with magnitude dependent noise can account for magnitude sensitivity. Our results are also in line with this account in which magnitude sensitivity is dependent upon magnitude sensitive noise in the decision process, and with other magnitude sensitive accounts such as the models inspired by magnitude sensitive decision dynamics in honeybee colonies [5, 6], or the sequential choice model [21]. Alternative diffusion models in which, given theoretically plausible reasons, magnitude manipulations are directly mapped onto parameters variations could also account for the type of results that we have observed here.

Evidence contrasting collapsing boundary explanations comes from the studies that have investigated this phenomenon in human perceptual and value-based decision making [12, 22, 23], using both large scale analysis of previously published data and newly ad-hoc investigations. Such studies have reported low-to-no support for collapsing boundaries in human decision making. Here we do not rule out the possibility that non-parallel decision making boundaries could provide an explanation for magnitude sensitivity for equal alternatives in decision making. However, we believe that the likelihood for such a scenario is low given that, to our knowledge, to date no studies have provided empirical or even theoretical support (with the exception of [11]) for non-parallel decision making boundaries. Furthermore, if simple models with fixed boundaries provide a better description compared to more complex parallel collapsing boundaries models [12, 22, 23] it is not clear why even more complex models, non-parallel collapsing boundaries, should provide a better description (i.e., a better trade-off between simplicity and goodness of fit) compared to parallel-collapsing or fixed boundaries.

The single-trial magnitude sensitivity that we observe cannot be explained by hypothesizing that subjects are actively adjusting their decision threshold during each trial in order to optimize reward rate for each single trial. Research has shown that the decision criterion is generally ‘slow’ to be actively adjusted, and it is generally set before stimulus appearance (see [4, 10]). In our case, the threshold adjustment at the single-trial level arises from the dynamics of the model itself, without any active commitment to adjust decision criteria. Although in our conceptualization of the LCA (a DDM with magnitude sensitive decision boundaries), magnitude sensitivity is described by threshold adjustment, in the LCA [9], magnitude sensitivity is generated spontaneously as a consequence of the lateral inhibition between evidence accumulators.

In our investigation we also found strong support for a difference between value and accuracy-based sessions in the relative speed that subjects had in order to make a decision, supporting a qualitative prediction for the difference between accuracy and value based decision making [11]. However, the DDM decomposition of data showed that in our case there was a difference between the intercept of the boundary separation between the accuracy-based and value-based sessions, but the magnitude-dependent slope did not differ. This result seems to suggests that while the baseline threshold varies between accuracy and value based session, the effect of single-trial magnitude on RTs does not differ between accuracy-based and value-based sessions. The result of faster decisions between accuracy and value-based instructions has similarities with how subjects adjust their decision criterion in accuracy compared to speed instructions [4]. This is a classical result in perceptual decision making, in which emphasizing speed over accuracy results in a decrease in boundary separation and in faster but more inaccurate responses. Overall, the result of faster and potentially less accurate decisions in value-based decision making compared to accuracy-based decision making can be attributed to the explanations already proposed [1–3]. Given that there is no correct or wrong response, faster but potentially inaccurate decisions that sacrifice small amount of accuracy, result in overall higher reward compared to the situation in which accuracy is stressed. This interpretation is also in line with theories of loss aversion [24] or avoidance of punishment [25] that could explain why subjects decrease their speed in order to increase accuracy in the accuracy based session.

If the result of behavioral performance deviating from the optimal account is corroborated by future investigation employing different stimuli and manipulations, an outstanding question for future research will be that of understanding why this is the case. For example future research could investigate whether single-trial magnitude sensitivity is due to biological constraints of the decision mechanism, as proposed by the multiplicative DDM account (see [2]). Another important question for future research could be investigating whether, by relaxing some of the assumptions made by the optimal account, it is possible to accommodate single-trial magnitude sensitive data within an optimal account. We believe that these questions could drive future fruitful projects.

## Conclusion

In conclusion, we show and corroborate evidence that magnitude sensitivity in decision making arises from single-trial dynamics in a bottom-up fashion. This result is incompatible with single-trial, statistically optimal, magnitude insensitive models, such as celebrated drift diffusion models.
